# Early Living Donor Liver Transplant Is Associated With Improved Graft‐ and Patient‐Survival Outcomes in Children With Biliary Atresia

**DOI:** 10.1111/petr.70367

**Published:** 2026-06-05

**Authors:** Marshall W. Wallace, Cameron M. Arkin, Anastasia M. Kahan, Chinedu Nwaduru, Francisca van der Schyff, Jean F. Botha, Zachary J. Kastenberg

**Affiliations:** ^1^ Division of Pediatric Surgery, Department of Surgery University of Utah Salt Lake City Utah USA; ^2^ Primary Children's Hospital, Intermountain Health Salt Lake City Utah USA; ^3^ Intermountain Medical Center, Intermountain Healthcare Salt Lake City Utah USA

**Keywords:** biliary atresia, liver transplant, living donor, living donor transplant

## Abstract

**Introduction:**

Biliary atresia (BA) is the most common indication for pediatric liver transplantation. Pediatric end‐stage liver disease (PELD) scores primarily determine deceased donor liver transplant (DDLT) waitlist priority. Due to persistent donor supply–demand mismatch, children typically have advanced disease and high PELD scores at the time of DDLT. Proactive living donor liver transplant (LDLT) may facilitate transplantation before critical illness. We hypothesized that early, low PELD, LDLT is associated with improved graft and patient survival in children with BA.

**Methods:**

We retrospectively reviewed children undergoing liver transplantation for BA in the SRTR database, comparing DDLT to LDLT, stratified by PELD. Early transplant was defined as transplant with a PELD ≤ 12. Four groups were analyzed: low PELD LDLT, low PELD DDLT, high PELD LDLT, and high PELD DDLT. Kaplan–Meier and Cox‐proportional hazards analyses evaluated patient‐ and graft‐survival.

**Results:**

4733 children meeting inclusion criteria were included (*n* = 249 Low PELD LDLT, *n* = 1152 Low PELD DDLT, *n* = 708 High PELD LDLT, *n* = 2624 High PELD DDLT). Prior to transplant, Low PELD LDLT recipients had lower INR and bilirubin levels, and required less life support (*p* < 0.01). Kaplan–Meier analyses showed the highest 5‐year graft and patient survival in the Low PELD LDLT (*p* < 0.01). Cox modeling revealed an independent association between low PELD LDLT and decreased graft loss (adjusted HR 0.45, *p* < 0.01) and mortality (adjusted HR 0.43, *p* = 0.03).

**Conclusions:**

A proactive approach to LDLT in children with BA is associated with improved long‐term graft and patient survival. Low PELD LDLT should be considered to increase early transplant access for children with BA.

## Graphical abstract

A proactive approach to living donor liver transplant in children with biliary atresia is associated with improved long‐term graft and patient survival. Low PELD living donor transplant should be considered to increase early transplant access for children with biliary atresia.

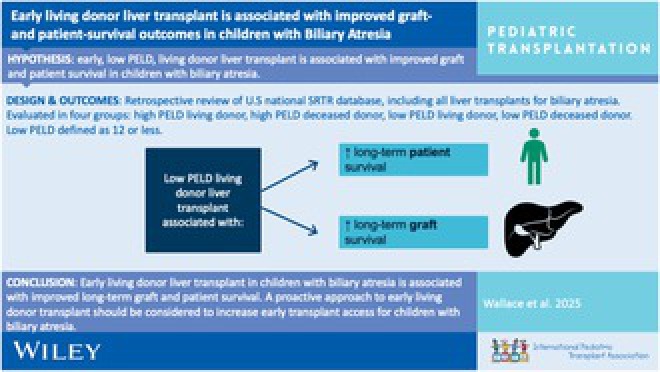

**Abbreviations**
BA: biliary atresia
DDLT: deceased donor liver transplant
ECMO: extracorporeal membrane oxygenation
HPE: hepatoportoenterostomy
LDLT: living donor liver transplant
OPTN: Organ Procurement and Transplantation Network
PELD: pediatric end‐stage liver disease
SRTR: Scientific Registry of Transplant Recipients
TVG: technical variant grafts

## 1 Background

Biliary atresia is the most common indication for pediatric liver transplantation [1]. In children with biliary atresia (BA), the current standard of care is the Kasai hepatoportoenterostomy (HPE) procedure, which aims to restore bile drainage and delay progression to end‐stage liver disease [2]. However, most patients will ultimately experience progressive liver failure despite HPE, ultimately necessitating liver transplantation [3, 4]. There is a shortage of deceased donor grafts, particularly for small children, leading to a relatively fixed number of annual pediatric deaths on the waiting list [1, 5]. The Pediatric End Stage Liver Disease (PELD) score is the primary means by which pediatric transplant candidates are prioritized for liver transplantation, with higher scores indicating more significant illness [6]. It has been shown that pre‐transplant life support and increasing PELD scores have been associated with decreased graft and patient survival in children with BA [7, 8].

Multiple strategies have been employed to address the supply versus demand mismatch, including expanding institutional donor acceptance criteria, the use of deceased donor technical variant grafts (TVG), and the use of living donor liver transplantation (LDLT) [9, 10, 11, 12]. Across multiple studies, LDLT is associated with improved survival outcomes compared to deceased donor liver transplant (DDLT) in children [13, 14, 15, 16].

Living donor transplantation allows for transplantation when the child is medically optimized (i.e., with low PELD scores and not yet requiring life support), eliminating the need to wait for a deceased donor organ.

LDLT has proven to be associated with improved patient survival compared to DDLT for patients with biliary atresia. Despite the proven benefit, outcomes stratified by severity of illness remain unknown [7]. There have been no prior multi‐center studies evaluating whether early LDLT, prior to the development of critical illness, is associated with improved graft or patient survival for children with BA. We hypothesized that early LDLT, defined by low PELD at the time of transplant for BA, would be associated with improved graft and patient survival.

## 2 Methods

### 2.1 Study Design and Population

After exemption approval by our institutional IRB (IRB #00176271), we performed a retrospective study of children undergoing liver transplantation for BA using data abstracted from the Scientific Registry of Transplant Recipients (SRTR). The SRTR data system includes data on all donors, wait‐listed candidates, and transplant recipients in the US, submitted by the members of the Organ Procurement and Transplantation Network (OPTN). The Health Resources and Services Administration (HRSA), U.S. Department of Health and Human Services provides oversight to the activities of the OPTN and SRTR contractors. The data reported here have been supplied by the Hennepin Healthcare Research Institute (HHRI) as the contractor for the Scientific Registry of Transplant Recipients (SRTR). The interpretation and reporting of these data are the responsibility of the authors and in no way should be seen as an official policy of or interpretation by the SRTR or the U.S. Government.

Data from pediatric patients (age 18 years or less) who underwent liver transplant between October 1987 (database inception) and March 2024 (time of data request) were abstracted from the SRTR [17]. Study inclusion criteria were age 18 years or less, diagnosis of BA (SRTR diagnosis code “4270: Biliary Atresia: Extrahepatic”) and liver transplant recipient status. Children with a history of prior liver transplant were excluded. Patients were analyzed in four cohorts based on PELD greater than or less than/equal to 12, and deceased or living donor liver transplant: Low PELD LDLT, Low PELD DDLT, High PELD LDLT, and High PELD DDLT. Arbitrary cutoffs of PELD 12 and 15 were initially analyzed, with equivalent results, so cutoff of PELD 12 was employed in this study. A sensitivity analysis was performed using Kaplan–Meier analyses, in which DDLT was separated into whole grafts and technical variant grafts (TVG), yielding 6 groups for analysis: Low PELD LDLT, Low PELD DDLT—whole graft, Low PELD DDLT –TVG, High PELD LDLT, High PELD DDLT—whole graft, High PELD DDLT –TVG. This study was performed in accordance with the Strengthening the Reporting of Observational Studies in Epidemiology (STROBE) guidelines.

### 2.2 Demographic and Transplant Variables

Data abstracted from the SRTR database included recipient age (months), recipient weight (kg), body mass index (BMI, kg/m^2^), gender, race, ethnicity, primary insurance payer, initial PELD score (not including exception points), exception point use, allocation score (PELD score + exception points), pre‐transplant serum sodium (mEq/L), international normalized ratio (INR), serum creatinine (mg/dL), serum SGPT (μ/L), total bilirubin (mg/dL), pre‐transplant ventilator use, pre‐transplant life support use (defined by the SRTR as any use of extracorporeal membrane oxygenation (ECMO) or ventricular assist devices, intravenous inotropes or mechanical ventilation dependency within 24 h prior to transplant), days on transplant waitlist, Status 1 listing (includes 1A and 1B), donor age (months), donor weight (kg), previous abdominal surgery (any previous non‐transplant abdominal surgery, to include, but not limited to, Kasai HPE), and cold ischemia time (hours).

To evaluate temporal patterns in pediatric liver transplantation for biliary atresia over time, we evaluated the cohort across three predefined eras: Era 1 was defined as any transplant before December 31, 1999, Era 2 was defined as any transplant between January 1, 2000 and December 31, 2009 and Era 3 was defined as occurring after January 1, 2010. Era 1 is the historical period, when pediatric liver transplant was primarily in its nascency and prior to the introduction of modern immunosuppression regimens. Era 2 is characterized by widespread introductions of modern immunosuppression agents such as tacrolimus, increased use of technical variant grafts, and the introduction of modern allocation protocols giving pediatric status 1 patients priority over adult status 1 patients [18, 19, 20]. Era 3 is the current era, characterized by increasing use of pediatric living donor liver transplant and broad practices focused on reduction of long‐term mortality in pediatric LT [1, 18, 21]. These era classifications closely mirror multiple similar recent publications evaluating temporal trends in liver transplant [18, 19, 21, 22].

### 2.3 Outcome Variables

Data abstracted from the SRTR database included hospital length of stay (days), graft and patient survival duration, and follow up interval. Graft loss was defined as any death or need for liver re‐transplant after receipt of a liver transplant. Hospital length of stay was defined as the duration of time from transplant to date of hospital discharge. Mortality was defined as any recorded date of death after liver transplantation. Follow up interval was defined as date of transplant to date of last follow up.

### 2.4 Statistical Analysis

Data were reported as medians with interquartile ranges for nonparametric continuous variables. Normality of the data was evaluated using the Shapiro–Wilk test and histogram visualization. Pearson's chi‐squared testing was used for categorical univariate analyses, while Wilcoxon rank sum testing was used for two‐group continuous univariate analyses. Missing continuous variable data was addressed using median imputation methods, with maximum imputation of 20%. Kaplan–Meier survival analyses were performed for patient and graft survival. Overall log‐rank testing was used to evaluate for significant differences in patient‐ and graft‐survival across the included groups within Kaplan–Meier models. Cox‐proportional hazard models were used to evaluate independent predictors of patient mortality or graft loss, using unadjusted and adjusted hazard ratios. Covariates for cox‐modeling were determined through a combination of evaluation of statistical significance in univariate analysis, and clinical significance, as determined by the study investigators. To select variables, all statistically significant (*p* < 0.05) variables between groups from Table 1 were considered. Principles of collinearity were then used in conjunction with clinical significance to select candidate variables while ensuring avoidance of multicollinearity (for example, recipient weight and age were not both included, though both significant. Similarly, INR or creatinine were not included, as they were already accounted for in the PELD score). As such, at least one variable from each subsection of Table 1 was included in the cox modeling, while simultaneously avoiding multicollinearity. For both Kaplan–Meier and Cox‐proportional hazards models, confirmed date of patient death, from death records incorporated into SRTR records, or date of graft loss, from SRTR records, were used to define the event of interest. Records were censored at time of latest follow‐up. Analyses were performed in R [23]. All tests were two‐sided, and *p*‐values set a priori at < 0.05 were considered statistically significant.

**TABLE 1:** Overall cohort description.

	Low PELD LDLT	Low PELD DDLT	High PELD LDLT	High PELD DDLT	*p*
	*N* = 249	*N* = 1152	*N* = 708	*N* = 2624	
Recipient demographics					
Age, months	16.0 (9.0 to 39.0)	22.5 (12.0 to 74.0)	8.0 (6.0–11.0)	10.0 (7.0–18.0)	< 0.01
Weight, kg	10.3 (8.1 to 16.5)	11.8 (8.6 to 21.3)	7.0 (5.9–8.2)	7.6 (6.4–9.7)	< 0.01
BMI (kg/m^2^)	16.9 (15.9 to 18.4)	17.1 (16.0 to 18.6)	16.9 (15.2–17.7)	16.9 (15.6–18.4)	< 0.01
Gender, male	109 (43.7%)	459 (39.8%)	285 (40.3%)	1016 (38.7%)	0.42
Race, White	166 (66.7%)	609 (52.8%)	474 (66.9%)	1433 (54.6%)	< 0.01
Non‐Hispanic	197 (79.1%)	869 (75.4%)	572 (80.7%)	2100 (80.0%)	0.01
Payer, private	145 (58.2%)	501 (43.5%)	425 (60.0%)	1092 (41.6%)	< 0.01
Recipient laboratory findings					
PELD	4 (−2 to 7)	5 (−1 to 9)	16 (16–24)	17 (16–24)	< 0.01
Na, mEq/L	137 (137 to 139)	137 (137 to 139)	137 (136–137)	137 (136–137)	< 0.01
INR	1.2 (1.1 to 1.3)	1.2 (1.1 to 1.3)	1.4 (1.4–1.8)	1.4 (1.4–1.8)	< 0.01
Cr, mg/dL	0.2 (0.2 to 0.3)	0.2 (0.2 to 0.3)	0.2 (0.2–0.2)	0.2 (0.2–0.3)	< 0.01
SGPT, μ/L	109 (109 to 117)	109 (90 to 117)	109 (109–109)	109 (109–109)	0.04
Bilirubin, mg/dL	2.1 (1.0 to 6.9)	2.4 (1.1 to 6.8)	10.8 (10.8–17.8)	10.8 (10.8–18.7)	< 0.01
Recipient preoperative clinical parameters					
Ventilator	2 (0.8%)	14 (1.2%)	25 (3.5%)	181 (6.9%)	< 0.01
Life support	2 (0.8%)	31 (2.7%)	34 (4.8%)	210 (8.0%)	< 0.01
Days on waitlist	105 (48 to 211)	112 (42 to 277)	69 (33–109)	78 (32–155)	< 0.01
Transplant/Listing parameters					
Status 1 A/B listing	2 (0.8%)	55 (4.7%)	33 (4.7%)	243 (9.3%)	< 0.01
Other Exception Score, *n*	76 (30.5%)	550 (47.7%)	153 (21.6%)	768 (29.3%)	< 0.01
Allocation score (PELD + exception)	38.0 (32.0 to 45.0)	33.0 (26.0 to 41.0)	59.0 (52.0–70.0)	58.0 (50.0–66.3)	< 0.01
Era of transplant					< 0.01
Era 1	1 (0.4%)	0 (0.0%)	196 (27.6%)	769 (29.3%)	
Era 2	83 (33.3%)	479 (41.6%)	203 (28.7%)	750 (28.6%)	
Era 3	165 (66.3%)	673 (58.4%)	309 (43.7%)	1105 (42.1%)	
Donor parameters					
Donor age, months	394 (344 to 458)	86 (25 to 185)	380 (317–437)	57 (17–188)	< 0.01
Donor weight, kg	70 (61 to 81)	25 (13 to 57)	61 (30–75)	18 (11–55)	< 0.01
Abd. surgery	216 (86.7%)	979 (84.9%)	561 (79.2%)	1984 (75.6%)	< 0.01
Cold ischemia, h	2.9 (1.0 to 4.0)	7 (5.6 to 8.7)	2.7 (1.3–6.7)	7.2 (6.0–9.2)	< 0.01
Deceased donor – Whole graft	N/A	783 (66.2%)	N/A	1632 (62.2%)	N/A
Deceased donor – Technical variant graft	N/A	369 (33.8%)	N/A	992 (37.8%)	N/A
Recipient clinical outcomes					
LOS, days	15 (10 to 20)	14 (9 to 22)	18 (13–29)	18 (12–31)	< 0.01
1‐year graft loss	11 (4.4%)	93 (8.1%)	58 (8.2%)	297 (11.3%)	< 0.01
3‐year graft loss	12 (4.8%)	98 (8.5%)	66 (9.3%)	330 (12.6%)	< 0.01
1‐year death	6 (2.4%)	39 (3.4%)	31 (4.4%)	154 (5.8%)	< 0.01
3‐year death	7 (2.8%)	44 (3.8%)	39 (5.5%)	187 (7.1%)	< 0.01
Follow up, years	6.0 (2.1 to 12.1)	8.2 (3.0 to 14.1)	8.1 (2.9–17.1)	9.0 (2.7–16.6)	< 0.01

*Note:* Total number included = 4733. Continuous data presented as median (IQR), categorical data presented as *n* (%). Era 1 (pre‐2000), Era 2 (2000–2009), Era 3 (2010 to present).

Abbreviations: DDLT, deceased donor liver transplant; LDLT, living donor liver transplant; PELD, pediatric end‐stage liver disease score.

## 3 Results

### 3.1 Overall Cohort Description

In total, 4733 children were included in the study, including 249 Low PELD LDLT, 1152 Low PELD DDLT, 708 High PELD LDLT, and 2624 High PELD DDLT. Of those undergoing Low PELD DDLT, 66.2% received a deceased donor whole graft. With respect to those undergoing High PELD DDLT, 62.2% received a deceased donor whole graft. The median PELD was 4 for the Low PELD LDLT group, 5 for Low PELD DDLT, 16 for High PELD LDLT, and 17 for High PELD DDLT (Table 1). The median pre‐transplant total bilirubin level was higher in the High PELD groups (10.8 mg/dL) compared to the Low PELD groups (2.1–2.4 mg/dL, *p* < 0.01). Low PELD patients were older and weighed more at the time of transplant compared to High PELD patients (Table 1). There were more white and privately insured recipients in the LDLT groups (Table 1).

### 3.2 Transplant Variables

Pre‐transplant ventilator use and life support were utilized at significantly higher rates in the High PELD groups, while the Low PELD patients spent longer on the wait list (Table 1). The High PELD DDLT group was most likely to be listed Status 1 (A or B) for transplant compared to the other groups (9.3% vs. 0.8%–4.7%, *p* < 0.01). Exception point use was highest in the Low PELD DDLT group (47.7% vs. 21.6%–30.5%, *p* < 0.01). Median allocation score (PELD + granted exception points) was highest in the High PELD LDLT group (59.0) and High PELD DDLT group (58.0) and lowest in the Low PELD LDLT group (38.0) and Low PELD DDLT group (33.0). Analyzed across three pre‐defined eras, relative use of living donor liver transplant grafts increased and deceased donor grafts decreased with time. In Era 1 (pre‐2020), 0.1% of Low PELD/LDLTs were performed and 27.6% of High PELD/DDLTs were performed, while in Era 3 (2010 to present), 66.3% of Low PELD/LDLTs and 43.7% of High PELD/LDLTs were performed. The median cold ischemia time was significantly longer with DDLT (~7 h) compared to LDLT (< 3 h, *p* < 0.01).

### 3.3 Transplant Outcomes

**FIGURE 1:**
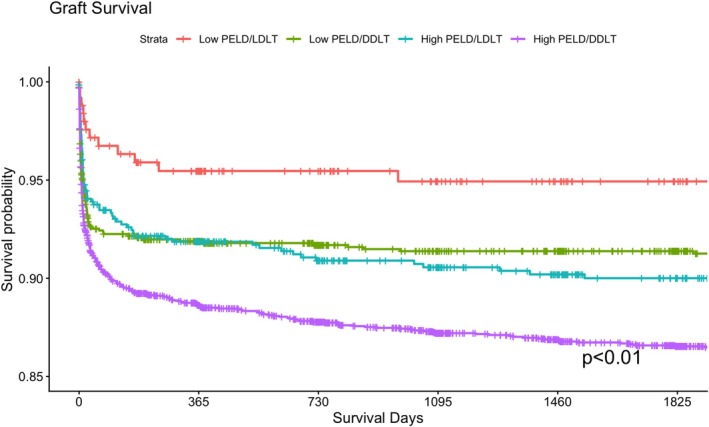
Graft‐survival, stratified by cohort low PELD/Deceased Donor (Low PELD/DDLT), Low PELD/Living Donor (Low PELD/LDLT), high PELD/deceased donor (High PELD/DDLT), High PELD/Living Donor (High PELD/LDLT).

**FIGURE 2:**
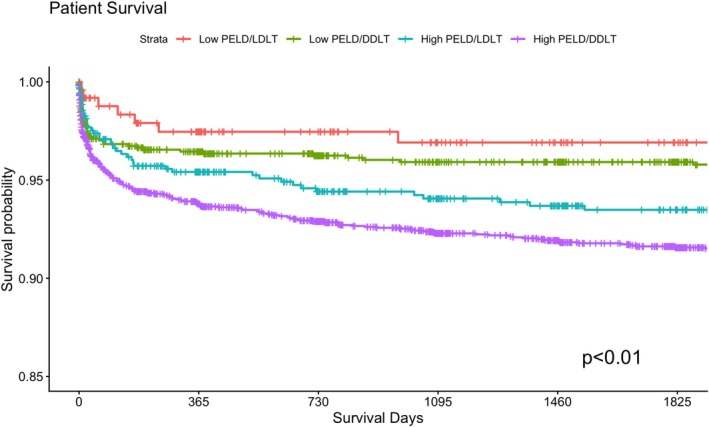
Patient survival, stratified by cohort Low PELD/Deceased Donor (Low PELD/DDLT), Low PELD/Living Donor (Low PELD/LDLT), High PELD/Deceased Donor (High PELD/DDLT), High PELD/Living Donor (High PELD/LDLT).

In univariate analysis, median hospital length of stay was significantly longer in the High PELD groups compared to the Low PELD groups (18 vs. 14–15 days, *p* < 0.01). Graft loss was lowest in the Low PELD LDLT group at 1 year (4.4% vs. 8.1%–11.3%, *p* < 0.01) and 3 years (4.8% vs. 8.5%–12.6%, *p* < 0.01). Similarly, mortality rates were lowest in the Low PELD LDLT group at 1 year (2.4% vs. 3.4%–5.8%, *p* < 0.01) and at 3 years (2.8% vs. 3.8%–7.1%, *p* < 0.01; Table 1). Kaplan–Meier survival analysis supported these findings, demonstrating significant differences across the four groups using overall log‐rank testing for both 5‐year graft survival (*p* < 0.01, Figure 1) and 5‐year patient survival (*p* < 0.01, Figure 2).

**FIGURE 3:**
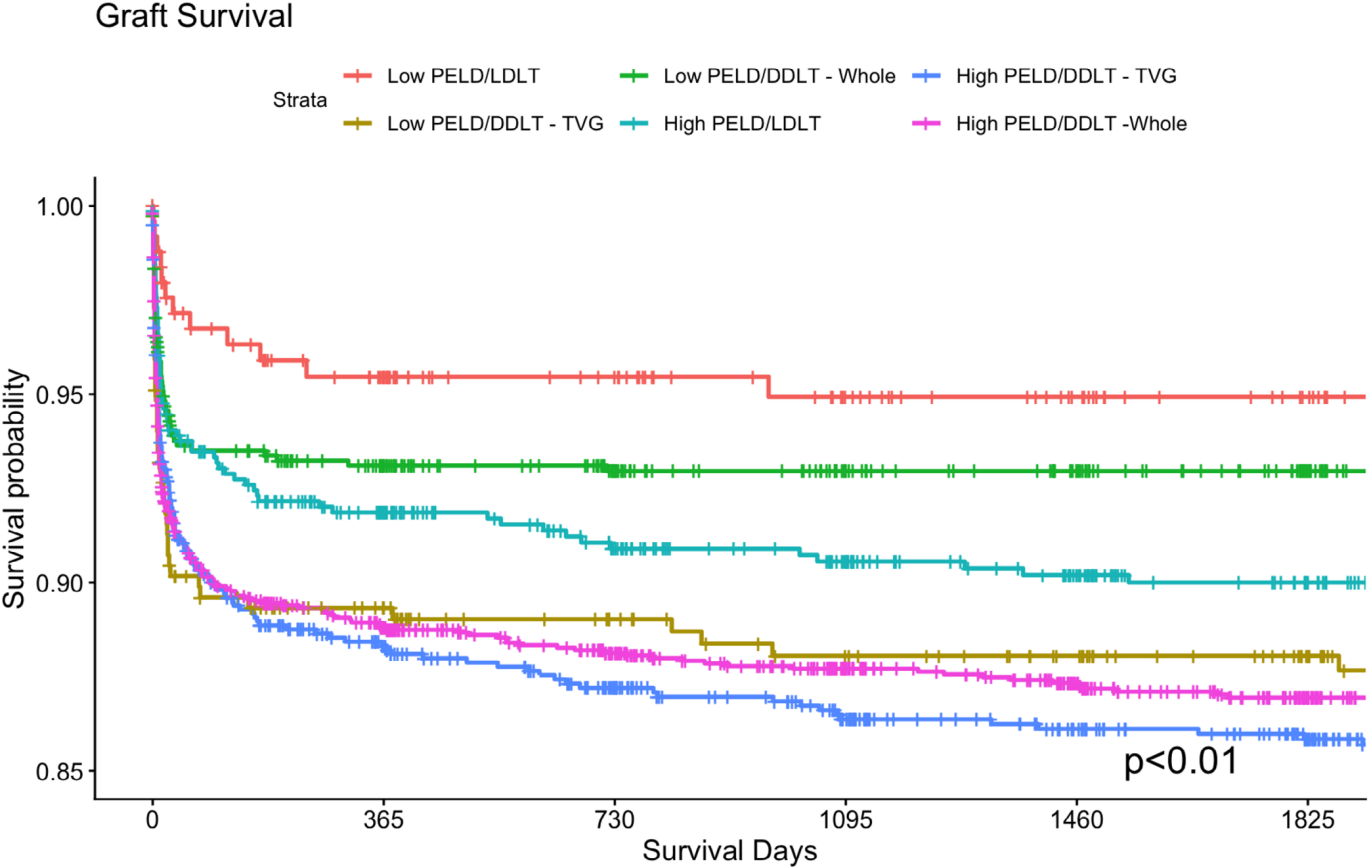
Graft‐survival, stratified by cohort Low PELD/Deceased Donor—Whole Graft (Low PELD/DDLT—Whole), Low PELD/Deceased Donor—Technical Variant Graft (Low PELD/DDLT—TVG), Low PELD/Living Donor (Low PELD/LDLT), High PELD/Deceased Donor—Whole Graft (High PELD/DDLT—Whole), High PELD/Deceased Donor—Technical Variant Graft (High PELD/DDLT—TVG), High PELD/Living Donor (High PELD/LDLT).

**FIGURE 4:**
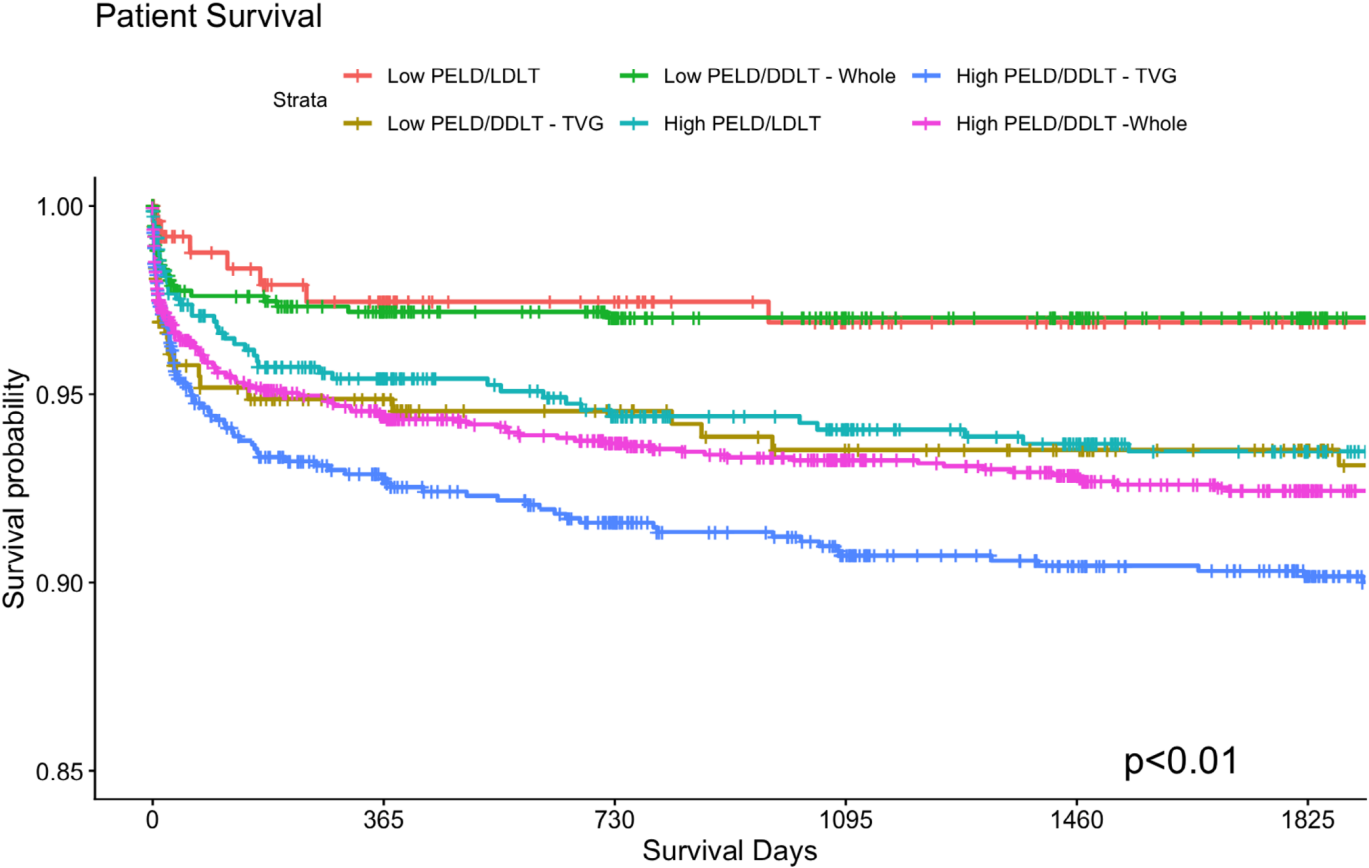
Patient‐survival, stratified by cohort Low PELD/Deceased Donor—Whole Graft (Low PELD/DDLT—Whole), Low PELD/Deceased Donor—Technical Variant Graft (Low PELD/DDLT—TVG), Low PELD/Living Donor (Low PELD/LDLT), High PELD/Deceased Donor—Whole Graft (High PELD/DDLT—Whole), High PELD/Deceased Donor—Technical Variant Graft (High PELD/DDLT—TVG), High PELD/Living Donor (High PELD/LDLT).

Kaplan–Meier survival analysis was repeated, evaluating the cohort in six groups (Low PELD LDLT, Low PELD DDLT—whole graft, Low PELD DDLT—technical variant graft (TVG), High PELD LDLT, High PELD DDLT—whole graft, High PELD DDLT—technical variant graft (TVG)). With respect to graft survival, Low PELD LDLT continued to have significantly higher rates of durable graft survival compared to all other groups (*p* < 0.01, Figure 3). With respect to patient survival, Low PELD LDLT and Low PELD DDLT—whole graft transplants appeared to have the highest patient survival compared to all other groups (*p* < 0.01, Figure 4). Across both 6‐way Kaplan–Meier analyses, DDLT—whole graft transplants appeared to have improved graft‐ and patient‐survival outcomes compared to DDLT—technical variant graft cohorts of the same PELD classification.

Unadjusted Cox Proportional Hazards modeling for graft loss and mortality (Tables 2 and 3) found sequential increase in hazards of both graft loss and mortality across PELD/donor categories with Low PELD LDLT recipients having the best outcomes (HR 0.34 for graft loss, *p* < 0.01; HR 0.31 for mortality, *p* < 0.01) with High PELD DDLT serving as the reference. In multivariable modeling (Tables 2 and 3), Low PELD LDLT recipients persisted as the group at lowest risk of both graft loss and death (HR 0.45 for graft loss, *p* < 0.01; HR 0.39 for mortality, *p* = 0.03). Importantly, cold ischemia time, pre‐transplant ventilator use, and Status 1 listing were independently associated with graft loss, mortality, or both in the multivariable models. Recipient weight and donor weight were not significant contributors following model adjustment.

**TABLE 2:** Cox proportional hazards model—graft loss.

Covariate	Unadjusted HR (95% CI)	*p*	Adjusted HR (95% CI)	*p*
Cohort				
High PELD/DDLT	Reference		Reference	
High PELD/LDLT	0.77 (0.61–0.97)	0.03	0.88 (0.66–1.17)	0.38
Low PELD/DDLT	0.71 (0.58–0.88)	< 0.01	0.81 (0.66–1.01)	0.06
Low PELD/LDLT	0.34 (0.19–0.59)	< 0.01	0.45 (0.25–0.83)	0.01
Recipient weight (kg)	0.99 (0.98–0.99)	< 0.01	0.99 (0.99–1.00)	0.18
Donor weight (kg)	0.99 (0.99–0.99)	0.02	1.00 (1.00–1.00)	0.81
Cold ischemia time (min)	1.04 (1.02–1.06)	< 0.01	1.03 (1.00–1.05)	0.02
Waitlist time (days)	0.99 (0.99–1.00)	0.02	1.00 (1.00–1.00)	0.12
Pre‐transplant ventilator	2.39 (1.83–3.12)	< 0.01	1.95 (1.49–2.61)	< 0.01
Status 1	1.79 (1.41–2.28)	< 0.01	1.32 (1.01–1.74)	0.04

*Note:* Results presented as unadjusted and adjusted hazard ratios (HR) and *p*‐values.

Abbreviations: DDLT, deceased donor liver transplant; LDLT, living donor liver transplant; PELD, pediatric end‐stage liver disease score.

**TABLE 3:** Cox proportional hazards model – patient mortality.

Covariate	Unadjusted HR (95% CI)	*p*	Adjusted HR (95% CI)	*p*
Cohort				
High PELD/DDLT	Reference		Reference	
High PELD/LDLT	0.82 (0.62–1.09)	0.18	0.95 (0.68–1.33)	0.765
Low PELD/DDLT	0.62 (0.47–0.81)	< 0.01	0.68 (0.51–0.90)	< 0.01
Low PELD/LDLT	0.31 (0.15–0.67)	< 0.01	0.39 (0.18–0.84)	0.02
Recipient weight (kg)	0.99 (0.98–1.01)	0.77	1.00 (0.99–1.01)	0.49
Donor weight (kg)	1.00 (0.99–1.01)	0.65	1.00 (1.00–1.01)	0.11
Cold ischemia time (min)	1.04 (1.01–1.06)	< 0.01	1.04 (1.01–1.06)	0.01
Waitlist time (days)	0.99 (0.99–1.00)	0.13	1.00 (1.00–1.00)	0.26
Pre‐transplant ventilator	1.75 (1.99–3.79)	< 0.01	2.30 (1.62–3.28)	< 0.01
Status 1	1.77 (1.32–2.39)	< 0.01	1.26 (0.89–1.78)	0.10

*Note:* Results presented as unadjusted and adjusted hazard ratios (HR) and *p*‐values.

## 4 Discussion

The aim of this study was to evaluate if early living donor liver transplant (LDLT), prior to critical illness and/or severe liver failure, was associated with improved graft and patient survival in children with BA. Using a comprehensive United States national transplant database, our study demonstrates that living donor liver transplantation in low‐PELD patients is independently associated with improved survival outcomes in children undergoing transplant for BA. These findings suggest that early, proactive living donor liver transplant is associated with long‐term benefits for children with BA and impending liver failure.

Our finding that early liver transplantation is associated with improved long‐term outcomes is in line with other recent, and remote, published work. Cowles et al. [24] evaluated a large single‐center experience with pediatric liver transplantation for BA in 2008 and found patient and graft survival to be 100% when transplant occurred at a PELD less than 10. LeeVan et al. [4] subsequently demonstrated improved long‐term survival with early primary liver transplantation compared to those undergoing Kasai HPE for BA. In the context of this literature, our study uniquely evaluates outcomes in early, low‐PELD, living and deceased donor transplants. Our findings are also in line with Montenovo et al.'s [13] recent study demonstrating LDLT's association with improved long‐term graft and patient survival compared to DDLT. Finally, our findings confirm and expand the report by Ziogas et al. [7] in which the authors demonstrated an association between improved patient survival and performance of LDLT in children with BA.

In this study, we demonstrated an association between low PELD LDLT and improvements in graft and patient survival outcomes. We used a PELD cutoff of 12 or less, which we selected to differentiate children with BA and impending hepatic failure from those with BA and ongoing hepatic failure, as liver transplant is ultimately anticipated in both groups. It is worth noting that both low PELD LDLT and low PELD DDLT groups in this study had pre‐transplant bilirubin levels greater than 2.0 mg/dL (medians 2.1–2.4 mg/dL), which is routinely used to indicate a non‐functioning Kasai HPE [25, 26]. As such, both LDLT groups in this study can be reasonably seen as candidates for liver transplantation and appropriate cohorts to evaluate the question of whether or not early LDLT is beneficial in BA.

Furthermore, in this study, the low PELD groups both had higher median weight at the time of transplant than the high PELD groups, highlighting that the term “early transplantation” is related to the progression of liver failure, not patient age or weight at the time of transplant. Importantly however, the Cox survival analyses for both graft failure and mortality showed that weight was not a significant predictor of transplant outcomes, suggesting that the potential for survival benefit with low PELD LDLT was not simply a reflection of older, larger children receiving LDLT, but rather children who are healthier at the time of transplant. Though not directly evaluated in this study, previous analyses have demonstrated a correlation between rates of pediatric living donor liver transplantation and improvements in center‐level wait list morbidity and mortality [27, 28, 29]. As such, a move toward increased consideration of low PELD LDLT in children with BA may benefit those children with BA, as suggested in this study's results, while also contributing to improvements in waitlist survival for children requiring liver transplantation overall.

Importantly, when evaluating patients undergoing DDLT‐TVG as a separate category, not surprisingly, DDLT—whole graft was associated with improved graft and patient survival compared to TVG. The main findings of this study, namely that low PELD LDLT patients fared better than their deceased donor and high PELD counterparts, held true in this sensitivity analysis. These findings support our conclusions that aggressive pursuit of living donor liver transplantation is appropriate for these children as there is not the same degree of a priori control over the type of deceased donor graft (whole vs. technical variant graft) as there is over donor selection in the time‐privileged scenario of the low PELD LDLT evaluation.

Children undergoing living donor transplantation, regardless of PELD score, were significantly more likely to be of white race and have private insurance as their primary payer. These findings suggest the potential for ongoing unmitigated disparities in LDLT for BA, and are in line with recent studies by Ebel et al., Mogul et al., and Wadhwani et al. [30, 31, 32] which report lower rates of LDLT in non‐white race children. Mogul et al. [32] also report lower use of LDLT in children with public health insurance. Future interventions that aim to increase multidisciplinary consideration of early LDLT in children with BA should carefully consider how to mitigate the potential for racial and insurance‐related disparities.

There are several limitations of this work worthy of discussion. First, this was a retrospective analysis of a large national database, with data analysis and abstraction performed at the level of individual institutions. Consequently, the conclusions drawn rely on the data collection fidelity at each institution and are limited to that which can be drawn from the data available in the database. For example, in this database, we can comment on the percentage of children with previous abdominal surgery, prior to transplant, but not on the rate of previous Kasai HPE, which though it is a common pre‐transplant surgery for children with BA, may increase the complexity of the transplant itself. Second, our study evaluated all data within the SRTR database across a 36‐year period. Pediatric hepatology, transplant and critical care have changed during the study period. Notably, across this period studied, donor selection and surgical technique have substantially changed with the introduction of TVG use and living donor graft use, modern‐era immunosuppression regimens have emerged (including tacrolimus), graft allocation rules have changed (including changes to UNOS listing status systems and prioritization of pediatric status 1 candidates) [18, 19, 20, 22]. Each of these may have varying effects on patient‐ and graft‐survival, and may thus have introduced time‐related unmeasured confounders to the outcomes evaluated. Third, this data lacked granularity regarding the specific clinical decision‐making influencing the timing of transplantation for individual patients. As such, we were unable to report on why one child may have received a liver transplant with a low PELD, while another did not receive transplant until reaching a higher PELD.

Similarly, this study compared candidates by natural PELD score, without incorporating allocation scores/exception points in the primary analysis. This strategy was selected because though exception point use is relatively high in liver transplant for BA, this measure is inherently less objective than the lab‐based natural PELD score [6, 33]. As such, comparing across using allocation scores as opposed to PELD could raise significant unmeasurable confounding. The rate of exception point use in this study, and within pediatric transplant broadly, raises questions regarding PELD's ability to adequately stratify pediatric liver transplant candidates, though the intricacies of this lie outside of the scope of this manuscript. Fourth, within the United States, the availability of LDLT, and frequency of LDLT performed, varies between pediatric transplant centers and regions [27]. As we did not evaluate center‐level outcomes, these practice variations may have led to unmeasured confounding. Finally, the choice to focus on liver transplant recipients limited our ability to evaluate what percentage of children with BA and low PELD (12 or less) would continue to thrive for years without transplantation. As some children with low PELD scores and BA may have ultimately not required liver transplantation, we may have introduced selection bias as these children would not have been included in the study. Further, the relative morbidity and mortality associated with remaining on the transplant wait‐list compared to undergoing early living donor liver transplant is not evaluated in this study of transplant recipients.

The practice‐informing associations identified in these data support proactive multidisciplinary discussion and consideration of LDLT for children with BA. Due to the time‐ and resource‐intensive nature of pediatric liver transplantation, particularly LDLT, these data further support ongoing efforts to improve early BA detection (such as routine neonatal screening and/or standardized 2 week evaluation of hyperbilirubinemic infants [34, 35]) and facilitate early referral of infants with direct hyperbilirubinemia for BA‐evaluation in a multi‐disciplinary liver transplant center. Specifically, the association between early, low PELD, transplant and improved post‐transplant outcomes underscores the importance of early multidisciplinary consideration of transplant options, including both living and deceased‐donor transplantation, in children with BA. This study has re‐demonstrated racial and socioeconomic disparities within living donor liver transplantation. To work toward improved equitable distribution of the scarce resource of living donor liver transplant, multidisciplinary evaluation and consideration of transplant listing should occur at referral centers that care for children with all forms of medical insurance coverage and evidence‐based measures such as anonymous non‐directed living donation and living donor liver transplant multi‐listing across centers should continue to be considered [36, 37].

In conclusion, this is the first study to evaluate graft and patient survival for low‐PELD LDLT in BA. In a time of clear supply–demand mismatch for deceased donor organs in pediatric transplant, LDLT may be associated with improved access to transplantation and long‐term improvements in graft and patient survival in affected children.

## Disclosure

The authors have nothing to report.

## Data Availability

The data that support the findings of this study are available in United States Scientific Registry of Transplant Recipients at https://www.srtr.org.
